# SARS-CoV-2 Spike Protein Induces Time-Dependent and Brain-Region-Specific Alterations in Ferroptosis Markers: A Preliminary Study in K18-hACE2 Mice

**DOI:** 10.3390/ijms27031526

**Published:** 2026-02-04

**Authors:** Asmaa Yehia, Chirine Toufaily, Dalia M. Abdel Ghaffar, Gehan El Wakeel, Mohamed Adel, Abeer F. Mostafa, Osama A. Abulseoud

**Affiliations:** 1 Department of Neuroscience, Graduate School of Biomedical Sciences, Mayo Clinic College of Medicine, Phoenix, AZ 58054, USA; 2 Department of Medical Physiology, Faculty of Medicine, Mansoura University, Mansoura 35516, Egypt; dalia.mr@mans.edu.eg (D.M.A.G.); madel@mans.edu.eg (M.A.); sara12@mans.edu.eg (A.F.M.); 3 Department of Basic Dental Sciences, Faculty of Dentistry, The Hashemite University, Zarqa 13115, Jordan; 4 Department of Medical Physiology, Faculty of Medicine, Mansoura National University, Gamasa 35712, Egypt; 5 Department of Psychiatry and Psychology, Mayo Clinic Arizona, Phoenix, AZ 85054, USA

**Keywords:** SARS-CoV-2, spike protein, post-COVID-19, ferroptosis, K18-hACE2

## Abstract

Post-COVID syndrome comprises persistent neuropsychiatric manifestations for more than 12 weeks after recovery from acute SARS-CoV-2 infection, yet its underlying pathophysiology is unclear. Ferroptosis, an iron-dependent form of cell death with three hallmarks, iron dysregulation, antioxidant failure, and lipid peroxidation, seems to be involved in COVID-19/post-COVID-19 pathophysiology. Here, we administered the SARS-CoV-2 spike protein S1 subunit intranasally to K18-hACE2 transgenic mice and quantified ferroptotic marker protein expression in four brain regions (hippocampus, prefrontal cortex, cerebellum, and olfactory bulb) at 2, 6, and 12 weeks post-administration, alongside ultrastructural assessment by transmission electron microscopy (TEM) that was limited to the hippocampus and prefrontal cortex. Two-way ANOVA revealed region- and time-dependent modulation of iron-handling, antioxidant, and lipid peroxidation markers. In the hippocampus, FPN1 was significantly increased at 2 weeks, while TFR1 showed a time-dependent pattern without significant week-specific differences. In the prefrontal cortex, DMT1 significantly increased at 2 weeks, and GPx4 showed an overall treatment effect with a trend of increase at 6 weeks. The cerebellum exhibited early increases in FPN1 and GPx4 and a delayed increase in MDA-conjugated proteins. In the olfactory bulb, FPN1 increased at 12 weeks, with GPx4 showing an overall treatment effect and an early trend of decrease. TEM identified ferroptosis-consistent features in the hippocampus and prefrontal cortex at all time points. These findings suggest that spike protein exposure may be associated with time-dependent and brain-region-specific alterations of ferroptosis-related markers. These preliminary findings are based on a limited sample size, which needs further research to elucidate the clinical implication and to study the mechanism in more depth as well as future validation with pharmacological inhibitors.

## 1. Introduction

**Figure 1:**
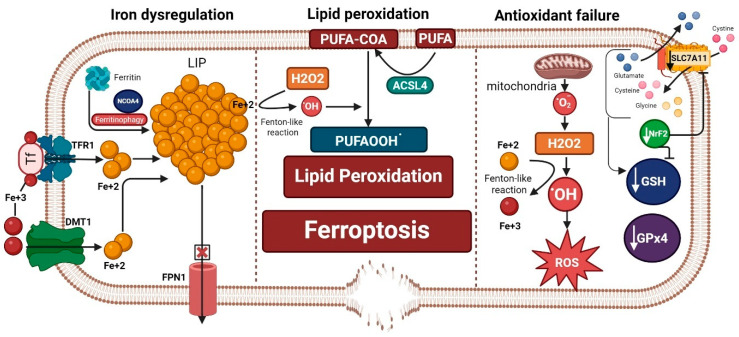
Ferroptosis machinery is composed of three hallmarks: iron dysregulation, antioxidant failure, and lipid peroxidation. Iron dysregulation, a driver of ferroptosis, occurs through increased labile iron pool (LIP). This can happen by release of iron from its storage sites in ferritin by ferritinophagy, increased iron import through transferrin receptor 1 (TFR1) and divalent metal transporter 1 (DMT1), and decreased iron export from the cell through ferroportin 1 (FPN1). Antioxidant failure occurs through increased ROS production that is fostered by increased LIP (ferrous iron: Fe^+2^) through a Fenton-like reaction. On the other hand, depletion of antioxidant defenses occurs by decreased glutathione (GSH), glutathione peroxidase 4 (GPx4), the main brakes of ferroptosis, and a decrease in the antioxidant transcription factor Nuclear Factor Erythroid 2-Related Factor 2 (NrF2). Decreased NrF2 leads to a reduction in GSH production and inhibits the cystine/glutamate antiporter SLC7A11/xCT. Slc7a11 is needed for exporting glutamate and importing cystine that is needed to form GSH along with glycine and glutamate after conversion to cysteine. Acyl-CoA synthetase long-chain family member 4 (ACSL4) incorporates long polyunsaturated fatty acids (PUFAs) into the cell membrane and converts them to PUFA-COA, which is crucial for the process of lipid peroxidation. Both iron dysregulation (through a Fenton-like reaction) and the production of hydrogen peroxide (H_2_O_2_) propagate the process of lipid peroxidation and the formation of more lipid peroxides (PUFAOOH^.^), which execute ferroptosis that leads to membrane disruption. “Created in BioRender. Hatem, A. (2026) https://BioRender.com/qzdemai, accessed on 28 December 2025”.

Ferroptosis is an iron-dependent form of cell death that was only discovered a decade ago [1]. The occurrence of ferroptosis has three prerequisites: first, iron dysregulation and accumulation of free labile iron capable of redox (oxidation-reduction) reactions and the production of reactive oxygen species (ROS); second, the failure of antioxidant lipid peroxide repair systems, especially the glutathione (GSH)–glutathione peroxidase 4 (GPx4) system, in the face of unopposed production of ROS; and third, lipid peroxidation and the production of lipid ROS, which is amplified by iron and failed antioxidant systems. Lipid peroxidation is considered the executor of ferroptosis, which ultimately produces highly toxic lipid peroxidation degradation products, such as malondialdehyde (MDA), that react with cell proteins and DNA, altering their structure and function and fostering a state of cytotoxicity. In addition, lipid peroxidation of membrane phospholipid polyunsaturated fatty acids (PUFAs) compromises the cell membrane integrity, leading to its thinning and rupture [1,2] (Figure 1).

Regulated ferroptosis plays a critical role in development, normal physiological aging, tumor suppression, and antiviral and overall immune function [3]. However, specific cellular pathophysiological mechanisms trigger uncontrolled ferroptosis in multiple neuropsychiatric disorders, such as depression and anxiety-like behavior [4,5], bipolar disorder [6], ischemic stroke [7,8], traumatic brain injury and its neuropsychiatric complications [9,10], Alzheimer’s [11,12,13], Parkinson’s [14,15], multiple sclerosis [16,17], amyotrophic lateral sclerosis [18,19], and possibly post-COVID syndrome [20].

Post-COVID syndrome, post-acute sequelae of Severe Acute Respiratory Syndrome Coronavirus type 2 (SARS-CoV-2) infection, and long COVID all refer to a group of neurological, cognitive, and psychiatric manifestations that persist for more than 12 weeks after recovery from acute SARS-CoV-2 infection regardless of illness severity and cannot be explained by an alternative diagnosis [21,22,23,24]. The most common manifestations include fatigue, anxiety, depression, headache, and cognitive impairment [25]. The magnitude of the post-COVID syndrome as a public health crisis cannot be underestimated. The incidence of post-COVID syndrome six months after recovery from acute infection among 236,379 patients was 33.6% (95% CI 33.17–34.07) and 46.4% (44.78–48.09) in patients who had been admitted to an intensive care unit [26]. A nationwide population cohort study of 198,096 Scottish adults estimated the prevalence of post-COVID syndrome to be 6.6–10.3% [27]. The pathophysiology of post-COVID syndrome remains under investigation [28]. However, all three elements of ferroptosis are documented in various studies.

Iron dysregulation serves as a protective mechanism during the acute illness since iron is a transition metal that acts as a catalyst for reactions that require electron transfer [29], such as the RNA-dependent RNA polymerase [30]. SARS-CoV-2 utilizes this iron-containing enzyme to replicate and synthesize its viral RNA. To deprive the virus of this essential element, the body sequesters iron into ferritin [31]. However, excessive iron storage into ferritin could be detrimental if it triggers nuclear receptor coactivator 4 (NCOA4)-mediated ferritinophagy [32]. Massive release of iron from ferritin generates oxidative stress and ferroptosis [20]. This state of acute iron dysregulation during COVID-19 illness manifests as high serum ferritin concentration and low serum iron content, signaling a worse outcome [33,34,35,36,37]. In addition, patients with moderate to severe COVID-19 who later developed post-COVID manifestations were found to have overexpression of monocyte iron homeostasis genes [38].

Failure of antioxidant systems is reported in acute SARS-CoV-2 infection in the form of glutathione (GSH) deficiency and a decrease in the total antioxidant capacity against the unopposed rise of oxidative stress [39,40,41,42,43]. GSH deficiency has been linked with COVID-19 severity and mortality [44]. In addition, SARS-CoV-2 directly suppresses mRNA expression of GPx4 [45] and indirectly affects its function by causing selenium deficiency, which was reported in COVID-19 patients and linked to higher mortality odds [46,47]. Selenium is integral for the function of GPx4, a selenoprotein that efficiently eliminates lipid hydroperoxides (lipid-ROS) [48]. These reports highlight an evident impact on both GSH and GPx4, the primary ferroptosis brakes [49], during COVID-19 acute infection. Nonetheless, the disruption of antioxidant defenses has been reported in post-COVID-19 patients, albeit less frequently. In a proof-of-concept study involving 120 post-COVID patients, Al-Hakeim et al. reported a significant correlation between post-COVID neuropsychiatric scores and reduced peripheral antioxidant defenses [50]. Using magnetic resonance spectroscopy, Saleh et al. detected lower levels of GSH in the frontal gray matter of 29 post-COVID patients, extending the finding of a failed antioxidant system to the brain [51]. Poletti et al. also depicted lower anterior cingulate cortex concentrations of GSH in 49 COVID-19 survivors, which are associated with increased depressive symptoms [52].

Lipid peroxidation biomarkers, including MDA and 4-hydroxynonenal (HNE-4), have been repeatedly linked with COVID-19 severity and mortality [53,54,55], besides a dysregulation of the lipid landscape [56,57]. These findings manifest in post-COVID-19 patients, where López-Hernández et al. found higher levels of several species of plasma phospholipid, phosphatidylcholines, and sphingomyelins in 20 post-COVID-19 patients compared to controls two years after recovery [58]. Also, Garrido et al. reported higher levels of other subclasses of plasma phospholipid, lysophosphatidylglycerols, and phosphatidylethanolamines in 147 post-COVID-19 patients up to 15 months after infection [59]. Despite these studies addressing plasma, not brain phospholipids, their findings could still be an argument for the occurrence of ferroptosis since the availability of phospholipid PUFA commands the sensitivity to ferroptosis [60,61]. This data collectively demonstrates the potential presence of ferroptosis as part of the COVID-19/post-COVID-19 pathophysiology.

To investigate changes in ferroptotic markers, we administered the SARS-CoV-2 spike protein S1 subunit intranasally to the K18-hACE2 transgenic mice at 8 weeks of age. We examined differences in protein expression levels of multiple ferroptotic markers (TRF1, DMT1, FPN1, NrF2, GPx4, and MDA-conjugated proteins) at four brain regions (hippocampus, prefrontal cortex, cerebellum, and olfactory bulb) at three time points: 2, 6, and 12 weeks post-S1 administration. We also examined ferroptotic features of subcellular structures in the hippocampus and prefrontal cortex using transmission electron microscopy (TEM) at the same three time points post-S1 administration. We used the spike protein and not the virus itself because administering the virus to ACE2 transgenic mice is lethal [62]. In addition, the spike protein alone directly impacts human cells [63], with one study showing that persistence of the spike protein up to 12 months was associated with post-COVID syndrome [64]. Furthermore, in animal models, the spike protein induces behavioral alterations reminiscent of post-COVID neuropsychiatric manifestations [65,66].

## 2. Results

### 2.1. Dynamic Protein Expression Changes of Ferroptosis Markers

#### 2.1.1. In the Hippocampus

**Figure 2:**
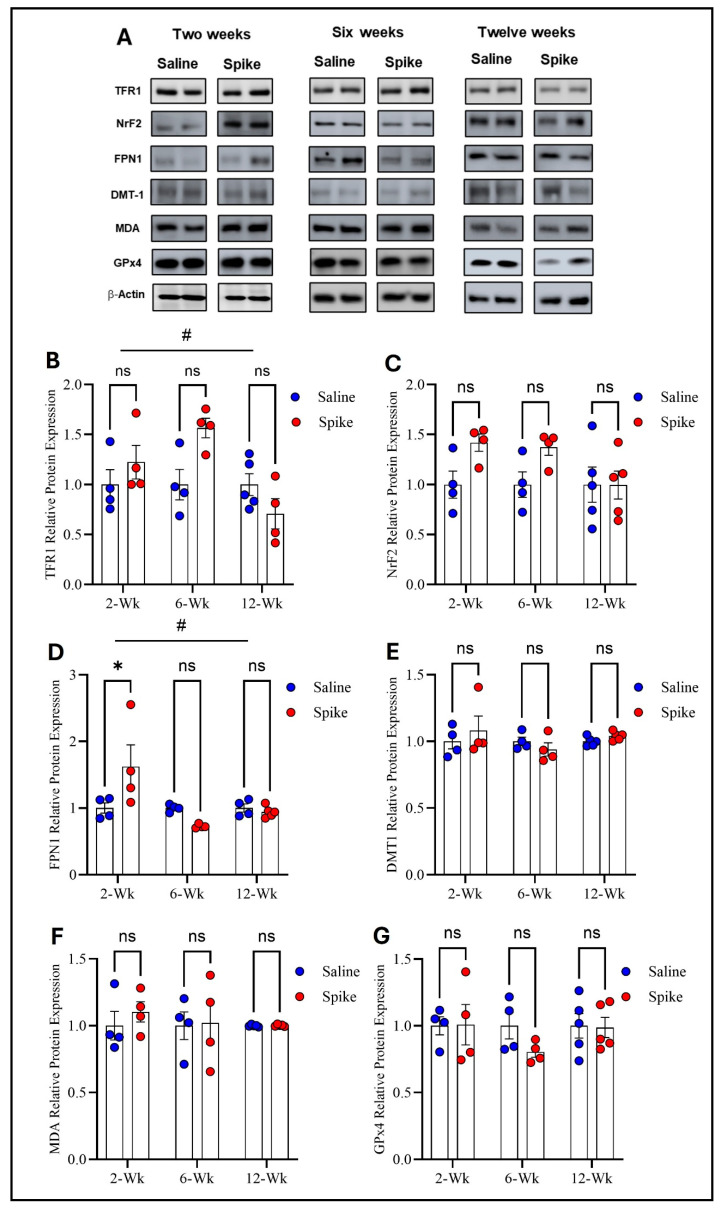
Changes in expression of ferroptotic markers in the hippocampus. Representative Western blot bands of measured ferroptosis markers and beta-actin are shown in (**A**). Two-way ANOVA identified a significant time effect # on TFR1 (F_1.584,15.05_ = 4.972, *p* = 0.028), but no statistically significant differences were observed in post hoc pairwise comparisons (**B**) or in FPN1 (time effect #: F_2,12_ = 6.879, *p* = 0.0102). Šídák post hoc comparisons showed a significant increase in FPN1 in the spike group at 2 weeks (adjusted *p* = 0.0126) (**D**); NRF2 (**C**), DMT1 (**E**), MDA-conjugated proteins (**F**), and GPx4 (**G**) showed no significant main effects or interactions. Data are represented as mean ± SEM; * *p* < 0.05. ns: non-significant.

Two-way ANOVA identified significant main effects of time on both TFR1 (F_1.584,15.05_ = 4.972, *p* = 0.028) and FPN1 protein expression (F_2,12_ = 6.879, *p* = 0.0102), as well as significant treatment × time interactions for TFR1 (F_2,19_ = 4.972, *p* = 0.0183) and FPN1 (F_2,12_ = 6.081, *p* = 0.015), indicating that expression of both proteins and the effect of S1 exposure differed across time points. No significant main effect of treatment was observed for either protein when averaged across all time points. Post hoc Šídák-adjusted comparisons showed no statistically significant differences between saline and spike groups for TFR1 at individual time points. In contrast, post hoc analyses for FPN1 demonstrated a significant increase in expression in the spike group at 2 weeks compared to controls (adjusted *p* = 0.013), with no significant differences detected at 6 or 12 weeks. In contrast to TFR1 and FPN1, no statistically significant effects were detected for DMT1, NrF2, MDA-conjugated proteins, or GPx4 protein expression. Two-way ANOVA identified no significant main effects of time or treatment or any significant treatment × time interactions for any of these markers (Figure 2 and Supplementary Table S1).

#### 2.1.2. In the Prefrontal Cortex

**Figure 3:**
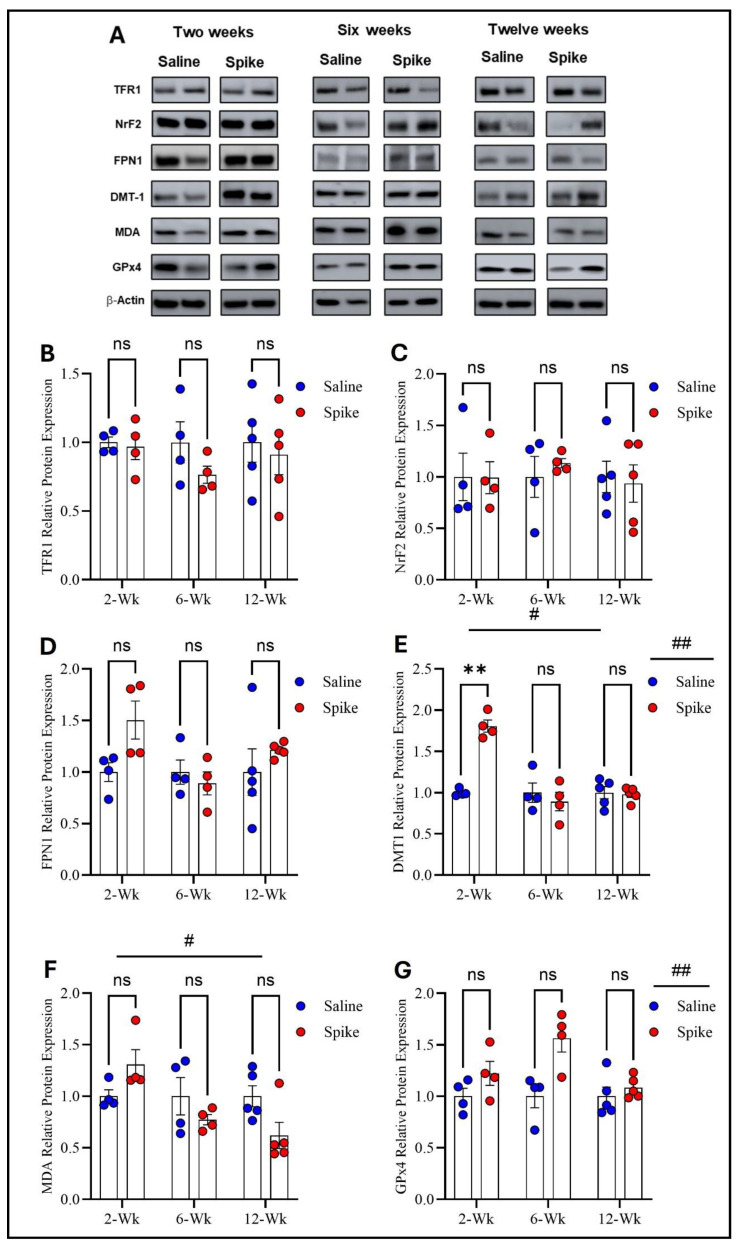
Changes in expression of ferroptotic markers in the prefrontal cortex. All Western blot representative bands of measured ferroptosis markers and beta-actin are shown in (**A**). Two-way ANOVA showed no significant main effects or interactions for TFR1 (**B**), NRF2 (**C**), or FPN1 (**D**). For DMT1, two-way ANOVA identified significant main effects of time # (F_1.541,15.41_ = 20.12, *p* = 0.0001) and treatment ## (F_1,20_ = 12.61, *p* = 0.002). Šídák post hoc testing demonstrated a significant increase in the spike group at 2 weeks (adjusted *p* = 0.002), with no differences at 6 or 12 weeks (**E**). For MDA-conjugated proteins, two-way ANOVA revealed a significant time effect # (F_1.858,11.15_ = 6.411, *p* = 0.015) (**F**). For GPx4, two-way ANOVA identified a significant main effect of treatment ## (F_1,20_ = 13.60, *p* = 0.001), with no statistically significant difference observed at any time point (**G**). Šídák post hoc comparisons were not significant (at 6 weeks, adjusted *p* = 0.052). Data are represented as mean ± SEM; ** *p* < 0.01, ns = non-significant.

Regarding TFR1, FPN1, and NrF2 protein expression, two-way ANOVA revealed no significant main effects of time or treatment or any significant interactions between the two factors. However, two-way ANOVA showed distinct patterns for DMT1, MDA-conjugated proteins, and GPx4 protein expression. For DMT1, two-way ANOVA identified significant main effects of time (F_1.541,15.41_ = 20.12, *p* = 0.0001) and treatment (F_1,20_ = 12.61, *p* = 0.002), as well as a significant treatment × time interaction (F_2,20_ = 20.12, *p* < 0.0001), indicating that DMT1 expression varied across time points and that the impact of S1 differed by time. Post hoc Šídák-adjusted comparisons demonstrated significantly higher DMT1 expression in the spike group at 2 weeks compared to controls (adjusted *p* = 0.002), with no significant differences at 6 or 12 weeks. For MDA-conjugated proteins, two-way ANOVA exhibited a significant main effect of time (F_1.858,11.15_ = 6.411, *p* = 0.015) and a significant treatment × time interaction (F_2,12_ = 6.712, *p* = 0.011), while the main effect of treatment was not significant. However, post hoc Šídák-adjusted comparisons did not identify significant differences between saline and spike groups at 2, 6, or 12 weeks. For GPx4, two-way ANOVA identified a significant main effect of treatment (F_1,20_ = 13.60, *p* = 0.001), with no significant main effect of time (F_1.994,19.94_ = 3.294, *p* = 0.058) and no significant interaction between the two factors (F_2,20_ = 3.294, *p* = 0.058). Post hoc Šídák-adjusted comparisons were not significant at any individual time points (Figure 3 and Supplementary Table S2).

#### 2.1.3. In the Cerebellum

**Figure 4:**
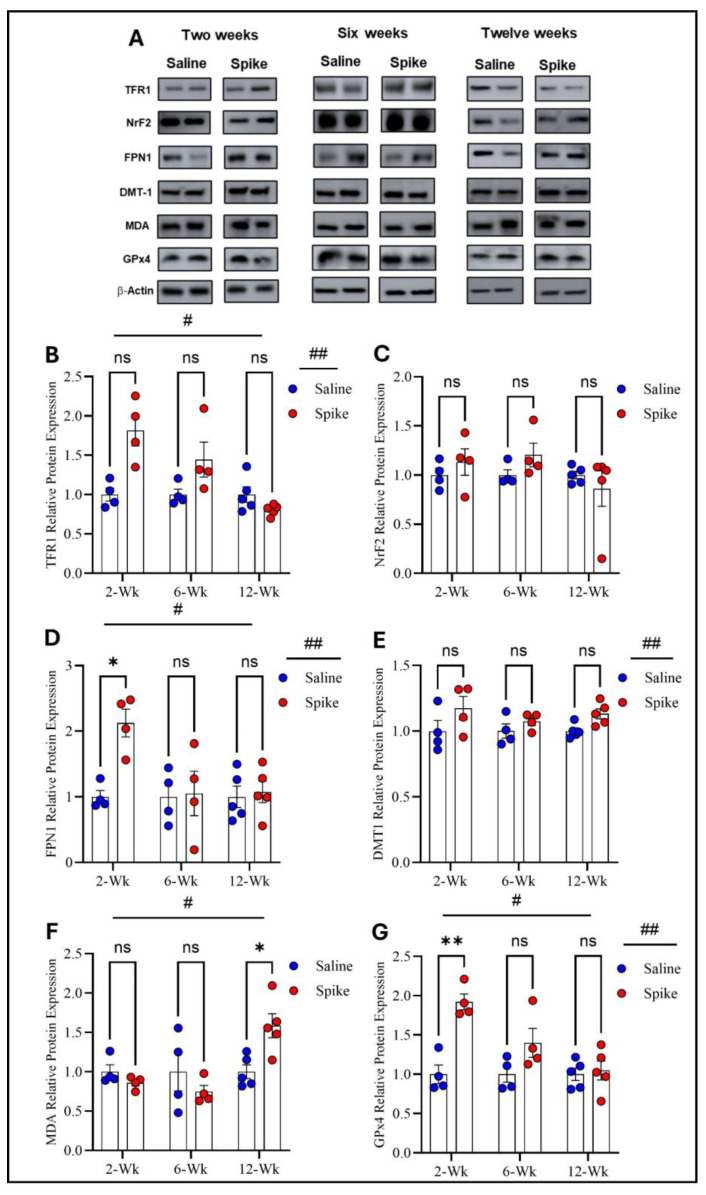
Changes in expression of ferroptotic markers in cerebellum. All Western blot representative bands of measured ferroptosis markers and beta-actin are shown in (**A**). Two-way ANOVA identified significant main effects of time # (F_1.654,16.54_ = 8.343, *p* = 0.0045) and treatment ## (F_1,20_ = 11.44, *p* = 0.003) for TFR1. Šídák post hoc comparisons were not significant at individual time points (at 2 weeks, adjusted *p* = 0.054) (**B**). NRF2 showed no significant main effects or interaction (**C**). For FPN1, two-way ANOVA showed significant main effects of time # (F_1.829,18.29_ = 4.339, *p* = 0.0315) and treatment ## (F_1,20_ = 6.266, *p* = 0.0211). Šídák post hoc testing demonstrated a significant increase in the spike group at 2 weeks (adjusted *p* = 0.0219), with no differences at 6 or 12 weeks (**D**). For DMT1, a significant main effect of treatment ## was detected (F_1,8_ = 6.296, *p* = 0.0364), with no significant time or interaction effects. Šídák comparisons were not significant (at 12 weeks, adjusted *p* = 0.0684) (**E**). For MDA-conjugated proteins, two-way ANOVA revealed a significant time effect # (F_1.830,10.98_ = 9.883, *p* = 0.004. Šídák post hoc testing identified a significant increase in the spike group at 12 weeks (adjusted *p* = 0.043) (**F**). For GPx4, two-way ANOVA showed significant main effects of time # (F_1.842,11.05_ = 15.17, *p* = 0.0008) and treatment ## (F_1,8_ = 12.47, *p* = 0.0077). Šídák post hoc comparisons demonstrated a significant increase in the spike group at 2 weeks (adjusted *p* = 0.0033), with no differences at later time points. (**G**). Data are represented as mean ± SEM; * *p* < 0.05, ** *p* < 0.01, ns = non-significant.

Two-way ANOVA identified significant main effects of time (F_1.654,16.54_ = 8.343, *p* = 0.004) and treatment (F_1,20_ = 11.44, *p* = 0.003) on TFR1 protein expression, as well as a significant treatment × time interaction (F_2,20_ = 8.343, *p* = 0.002). However, post hoc Šídák-adjusted comparisons between saline and spike groups at 2, 6, and 12 weeks did not reach statistical significance. For FPN1, two-way ANOVA also showed significant main effects of time (F_1.829,18.29_ = 4.339, *p* = 0.03) and treatment (F_1,20_ = 6.266, *p* = 0.02), with significant treatment × time interaction (F_2,20_ = 4.339, *p* = 0.027). Post hoc analyses exhibited significantly higher FPN1 expression in the spike group at 2 weeks compared to controls (adjusted *p* = 0.021), with no significant differences at 6 or 12 weeks. For DMT1, a significant main effect of treatment was detected (F_1,8_ = 6.296, *p* = 0.036), while the main effect of time and the treatment × time interaction were not significant. Post hoc comparisons did not identify significant differences at individual time points. NrF2 showed no significant main effects or treatment × time interaction by two-way ANOVA and no significant post hoc differences at 2, 6, or 12 weeks. For MDA-conjugated proteins, two-way ANOVA revealed a significant main effect of time (F_1.830,10.98_ = 9.883, *p* = 0.004) and a significant treatment × time interaction (F_2,12_ = 10.74, *p* = 0.0021), while the main effect of treatment was not significant. Post hoc analyses identified a significant increase in MDA-conjugated protein expression in the spike group at 12 weeks (adjusted *p* = 0.043), with no significant differences at earlier time points. Finally, GPx4 exhibited significant main effects of time (F_1.842, 11.05_ = 15.17, *p* = 0.0008) and treatment (F_1,8_ = 12.47, *p* = 0.0077), along with significant treatment × time interaction (F_2,12_ = 14.87, *p* = 0.0006). Post hoc Šídák-adjusted comparisons demonstrated significantly higher GPx4 expression in the spike group at 2 weeks (adjusted *p* = 0.0033), with no significant differences at 6 or 12 weeks (Figure 4 and Supplementary Table S3).

#### 2.1.4. In the Olfactory Bulb

**Figure 5:**
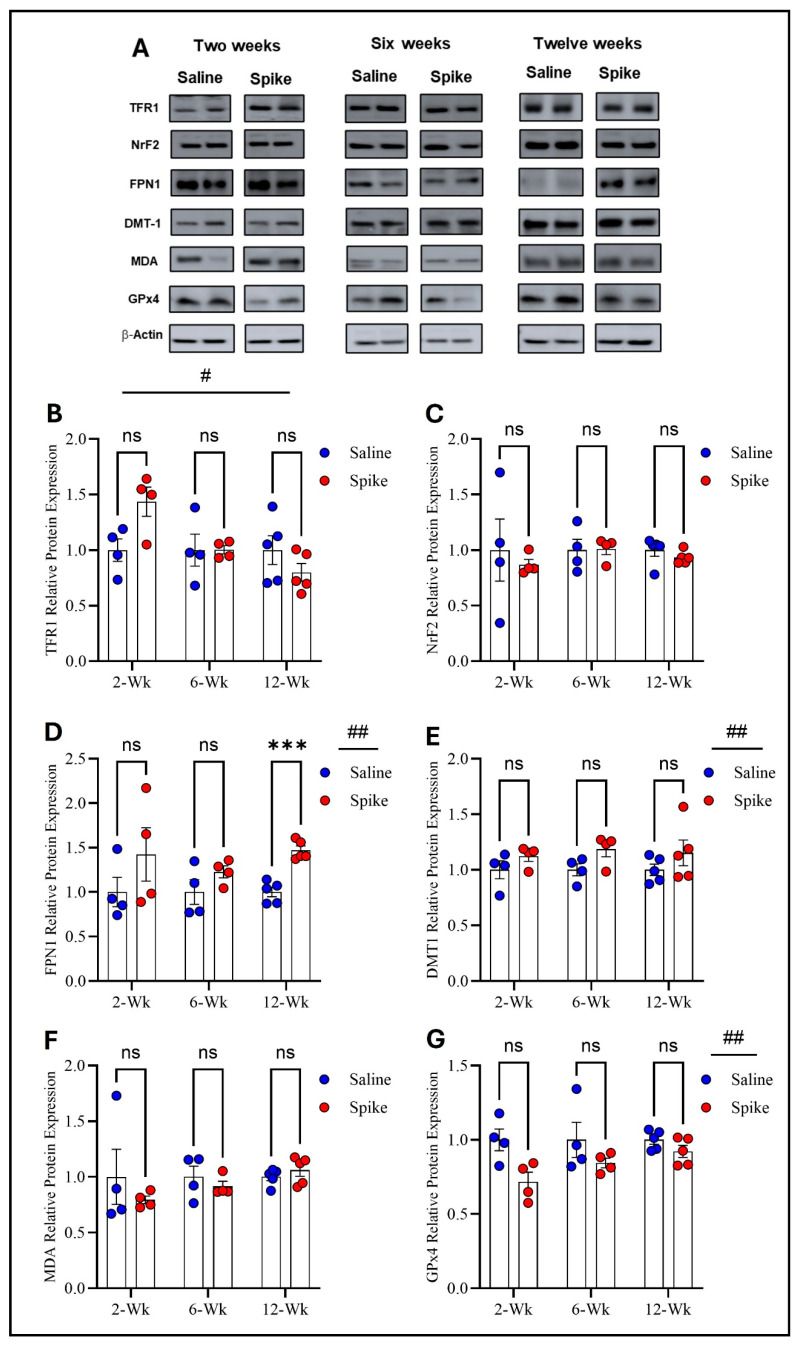
Changes in expression of ferroptotic markers in the olfactory bulb. All Western blot representative bands of measured ferroptosis markers and beta-actin are shown in (**A**). Two-way ANOVA identified a significant main effect of time # for TFR1 (F_1.998,11.99_ = 11.93, *p* = 0.001), with no significant main effect of treatment. Šídák post hoc comparisons between saline and spike groups were not significant at 2, 6, or 12 weeks (**B**). For FPN1, two-way ANOVA showed a significant main effect of treatment ## (F_1,8_ = 8.329, *p* = 0.02) with no significant time or interaction effects. Šídák post hoc testing identified a significant increase in the spike group at 12 weeks (adjusted *p* = 0.0006) (**D**). For DMT1, a significant main effect of treatment ## was detected (F_1,20_ = 5.962, *p* = 0.024). Šídák comparisons were not significant at individual time points (**E**). NRF2 (**C**) and MDA-conjugated proteins (**F**) showed no significant main effects or interactions. For GPx4, two-way ANOVA revealed a significant main effect of treatment ## (F_1,8_ = 8.051, *p* = 0.021) with no significant time or interaction effects. Šídák post hoc comparisons were not significant (at 2 weeks, adjusted *p* = 0.0799) (**G**). Data are represented as mean ± SEM; *** *p* < 0.001, ns = non-significant.

Two-way ANOVA identified a significant main effect of time (F_1.998,11.99_ = 11.93, *p* = 0.001) on TFR1 expression and a significant treatment × time interaction (F_2,12_ = 8.72, *p* = 0.004), while no significant main effect of treatment was identified. Post hoc Šídák-adjusted comparisons between saline and spike groups at 2, 6, and 12 weeks were not significant. For FPN1, a significant main effect of treatment was observed (F_1,8_ = 8.329, *p* = 0.02), whereas the main effect of time and the treatment × time interaction were not significant. Post hoc analyses identified a significant increase in FPN1 in the spike group at 12 weeks (adjusted *p* = 0.0006), with no significant differences at 2 or 6 weeks. For DMT1, two-way ANOVA also detected a significant main effect of treatment (F_1,20_ = 5.962, *p* = 0.024), with no significant main effect of time or treatment × time interaction and no significant post hoc differences at individual time points. NrF2 and MDA-conjugated proteins showed no significant main effects of time, treatment, or treatment × time interactions by two-way ANOVA and no significant post hoc differences at any time point. Finally, GPx4 exhibited a significant main effect of treatment (F_1,8_ = 8.051, *p* = 0.021) with no significant main effect of time or treatment × time interaction. Post hoc Šídák-adjusted comparisons were not significant at individual time points (Figure 5 and Supplementary Table S4).

**Figure 6:**
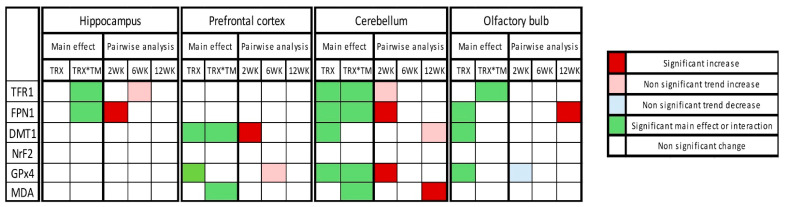
Heatmap of changes in expression of ferroptotic markers in tested four brain regions at three time points after SARS-CoV-2 spike protein administration. Heatmap summarizes the two-way ANOVA results, including treatment (TRX) main effect and treatment × time interactions (TRX*TM) and post hoc Šídák-adjusted pairwise analysis.

The overall summary of the results is demonstrated in Figure 6.

### 2.2. Ferroptosis Features Detected by Transmission Electron Microscopy

**Figure 7:**
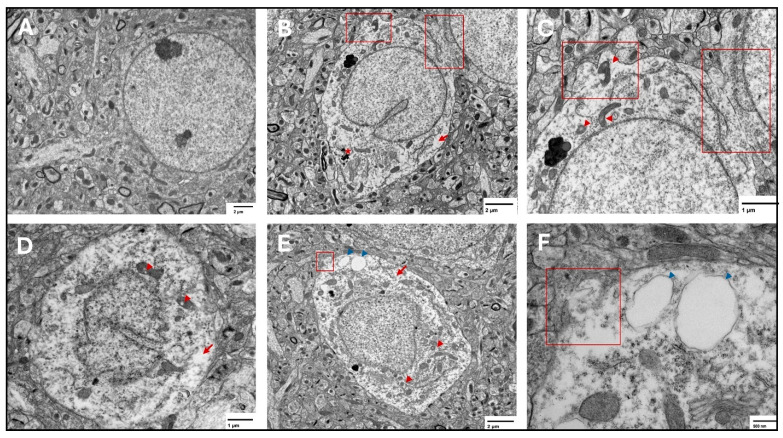
Representative transmission electron microscopy images of ferroptotic features in prefrontal cortex neurons. Representative images from the saline group show normal neurons with normal nuclear and cytoplasmic lucency and no evident plasma membrane disruption (**A**). Representative images from the two-week spike group (**B**,**C**) show disrupted plasma membrane (red squares), magnified in (**C**), translucent cytoplasm (arrows), and shrunken mitochondria with disrupted cristae (arrowheads) in (**C**), electron dense non-uniform deposition appears in (**B**) (*). Representative images from the six-week spike group (**D**) show disrupted mitochondrial cristae (arrowheads) and translucent cytoplasm (arrow). Representative images from the twelve-week spike group (**E**,**F**) show plasma membrane disruption (magnified in (**F**), red square), translucent cytoplasm (red arrow), shrunken mitochondria (red arrowheads), and cytoplasmic vacuolation (blue arrowheads, magnified in (**F**)).

**Figure 8:**
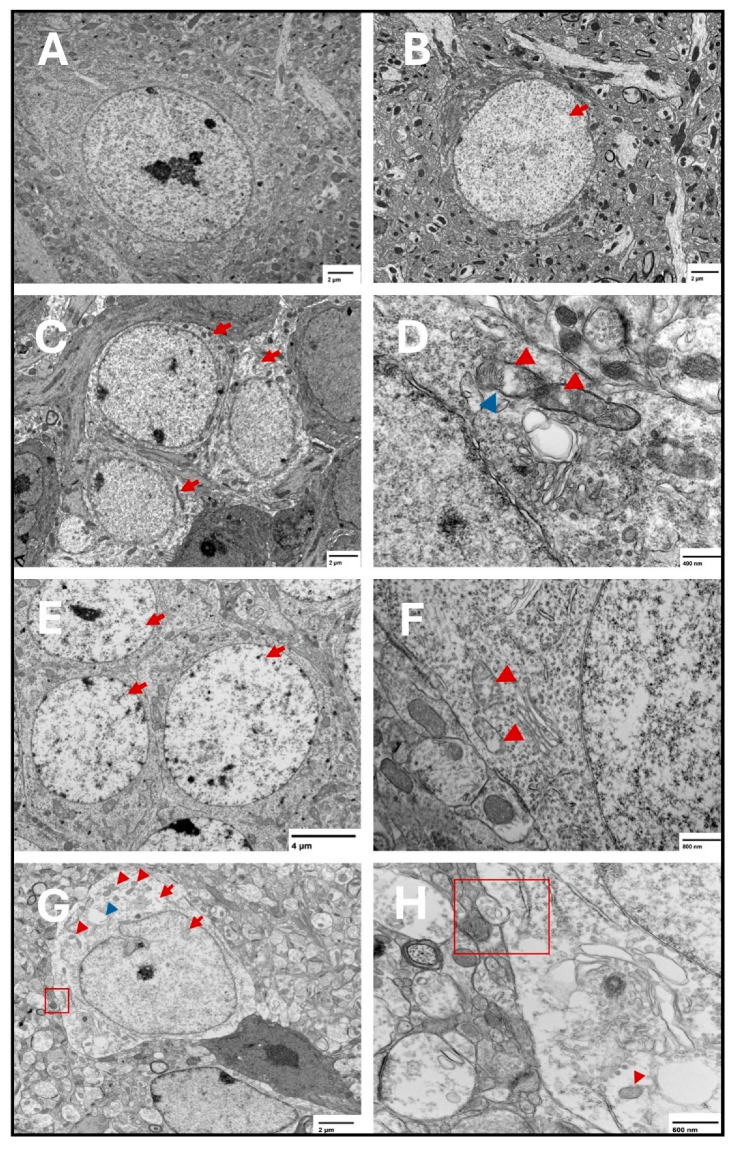
Representative transmission electron microscopy images of ferroptotic features in hippocampal neurons. Representative images from the saline group show normal neurons with normal nuclear and cytoplasmic lucency and no evident plasma membrane disruption (**A**). Representative images from the two-week spike group show translucent nucleus (**B**) (arrow). Electron lucent cytoplasm is shown in (**C**) (arrows) in the two-week spike group. In addition, representative images from the two-week spike group show disrupted mitochondrial cristae (**D**) (red arrowheads) and ruptured outer mitochondrial membrane (**D**) (blue arrowhead). Representative images from the six-week spike group show translucent nuclei in multiple neurons (**E**) (arrows) and disrupted mitochondrial cristae (**F**) (arrowheads). Representative images from the twelve-week spike group show plasma membrane disruption ((**G**): magnified in (**H**)) (red square), translucent cytoplasm and nucleus (**G**) (red arrows), shrunken mitochondria ((**G**): magnified in (**H**)) (red arrowheads), and cytoplasmic vacuolation (**G**) (blue arrowhead).

At the three tested points, features of ferroptosis were observed in the prefrontal cortex (Figure 7) and hippocampus (Figure 8), including electron-lucent nuclei and cytoplasm, cell membrane disruption, and mitochondrial changes that included shrunken mitochondria, disrupted mitochondrial cristae, and ruptured outer membranes.

## 3. Discussion

The present study demonstrates that a single intranasal (IN) administration of SARS-CoV-2 spike protein S1 to K18-hACE2 transgenic mice elicits a persistent molecular response in ferroptosis-related markers, alongside ultrastructural features consistent with ferroptotic injury in the hippocampus and prefrontal cortex [3,67]. This response is both time-dependent and brain-region-specific, which is shown in the olfactory bulb, prefrontal cortex, hippocampus, and cerebellum, where a distinct statistical pattern was observed across regions and tested time points.

In the hippocampus, significant treatment × time interactions were identified for both TFR1 and FPN1, indicating a time-dependent treatment influence on iron-handling markers. FPN1 showed a significant increase at 2 weeks in the spike group, while TFR1 showed a trend toward higher expression in the spike group at 6 weeks. These findings suggest that hippocampal iron-export machinery may be engaged early after S1 exposure (increased FPN1), while TFR1 changes may be more temporally related rather than dominated by a single time point. Notably, hippocampal DMT1, NrF2, GPx4, and MDA-conjugated proteins did not show statistically significant effects, indicating that in the hippocampus and within the tested markers, iron-handling shifts were more prominent than detectable antioxidant or lipid peroxidation markers across the three time points. Dysregulation of iron-related markers aligns with the hippocampus high metabolic demand and iron content, which may disrupt hippocampal synaptic integrity and neuronal viability, potentially contributing to memory deficits and cognitive impairment reported following COVID19 infection [68,69].

In the prefrontal cortex, TFR1, FPN1, and NrF2 did not show significant time, treatment, or interaction effects. In contrast, DMT1 showed higher expression in the spike group at 2 weeks as well as a robust time and treatment effects with a significant treatment × time interaction. This pattern supports early enhancement of iron-import capacity in the prefrontal cortex following S1 exposure. Additionally, significant time and treatment × time effects for MDA-conjugated proteins, without significant post hoc differences at individual time points, suggest temporally related impact of S1 on oxidative damage that may not be strongly localized to a single time point after multiplicity adjustment. Finally, GPx4 showed a significant main effect of treatment, with a non-significant trend of increased expression at 6 weeks. Together, these results indicate that in the prefrontal cortex, S1 exposure is accompanied by early iron-transport modulation (via DMT1) and treatment-associated differences in GPx4, consistent with engagement of antioxidant defenses.

The cerebellum showed the most consistent evidence for time-dependent modulation across multiple ferroptosis-related markers. Tested iron-related markers, TFR1, FPN1, and DMT1 showed significant treatment effects, while only TFR1 and FPN1 showed significant treatment × time interaction. No statistically significant differences were observed for TFR1 between the spike and saline groups at any time point. FPN1 showed a clear post hoc increase at 2 weeks. Similarly, DMT1 did not show statistically significant differences at individual time points. Importantly, cerebellar MDA-conjugated proteins showed significant interaction effects, with a significant increase in the spike group at 12 weeks, pointing to late-emerging oxidative damage signal in the cerebellum. In parallel, GPx4 displayed a significant treatment effect, significant interaction, and a significant post hoc increase at 2 weeks. Taken together, the cerebellum demonstrates an early pattern compatible with compensatory antioxidant engagement (increased GPx4 and increased FPN1 suggesting iron export) alongside later evidence of lipid peroxidation-associated damage (increase in MDA-conjugated proteins at 12 weeks). This temporal change suggests early protective responses that may be insufficient to prevent later oxidative injury under persistent or evolving stressors, suggesting delayed ferroptotic vulnerability in this region. This delayed vulnerability in the cerebellum could manifest as subtle motor or coordination deficits, which is reported in post-COVID-19 patients [70]. However, the upregulation of GPx4 is inconsistent with the classic ferroptotic profile of antioxidant failure. In addition, the lack of TEM assessment of cerebellar ferroptotic features renders the data in the cerebellum to most likely indicate cellular stress and compensatory resistance, rather than the execution of ferroptosis.

In the olfactory bulb, TFR1 demonstrated a significant treatment × time interaction but without significant post hoc differences at individual time points. FPN1 showed a significant main effect of treatment and a significant post hoc increase at 12 weeks, suggesting altered iron export capacity at later stages. DMT1 showed a significant main effect of treatment without significant post hoc week-specific differences. NrF2 and MDA-conjugated proteins did not show significant effects. GPx4 displayed a significant main effect of treatment. Collectively, these findings suggest that the olfactory bulb displays treatment-associated shifts in iron-handling (notably FPN1 at 12 weeks). These changes imply delayed olfactory bulb vulnerability after S1 administration. The treatment-related effect of the spike protein on multiple ferroptotic markers in the olfactory bulb could underline the olfactory dysfunction, which is reported to persist in long-COVID conditions [71,72].

Notably, the measured markers are heavily implicated in ferroptosis machinery. TFR1 elevated expression is a strong indication of ferroptosis since TFR1 is considered to be a specific ferroptosis marker that can even increase sensitivity to ferroptosis [73,74,75,76]. Interestingly, TFR1 was found to act as an alternative receptor for the SARS-CoV-2 spike protein to allow cellular viral entry [77]. In addition, DMT-1 overexpression emphasizes ferroptotic changes as it acts as a ferroptosis driver in early brain injury as well as in other contexts [78,79,80,81,82]. GPx4, a ferroptosis brake, displayed region-specific favoritism, where it increased in the cerebellum and decreased in the olfactory bulb at the same time (two weeks). This could indicate an earlier vulnerability of the olfactory bulb and room for compensatory mechanisms in the cerebellum [83]. Furthermore, MDA-conjugated proteins not only mark lipid peroxidation occurrence but also indicate that MDA formed covalent adducts on proteins and caused oxidative lipid-derived protein damage [84].

Notably, interaction effects without strong week-specific post hoc contrasts for certain markers suggest that some treatment influences manifest as distributed temporal shifts rather than isolated peaks at one time point.

Our in vivo results add to the current literature which shows that the spike protein induces ferroptosis in different cell types [THP-1-derived macrophages [85], Huh7 and Calu-3 cells [83], and H9C2 cells [86]]. Here we show that a single dose of the spike protein administered intranasally to the K18-hACE2 transgenic mice induces long-lasting protein expression changes in several ferroptotic and anti-ferroptosis markers in different brain regions. The K18h-ACE2 mice have been used to model COVID-19 infection [87,88] through the IN administration of the SARS-CoV-2 virus itself. However, the mortality rate after IN viral administration was high, which did not allow for the disease progression to be studied [89,90,91]. Intracerebroventricular administration of the SARS-CoV-2 spike protein has been used to model post-COVID syndrome but not in K18-hACE2 mice [65]. Rhea et al. assessed whether the SARS-CoV-2 spike protein reaches the brain by intravenous (IV) and IN administration. They reported that the SARS-CoV-2 spike protein reaches the brain at a much lower concentration by IN compared to IV administration [92]. However, they used a dose of 12.5 ng/mouse, while we used a dose of (0.6 μg/g, or 12 μg/20g mouse). We used the S1 subunit of the SARS-CoV-2 spike protein based on iterative reports of how it is implicated in the pathogenesis of post-COVID syndrome [93]. The full-length spike protein is composed of two subunits, S1 and S2, with a furin cleavage site (FCS). S1 must be first cleaved by host cell protease to allow binding to the ACE2 receptor and viral entry into the cell [94]. Therefore, the use of K18-hACE2 mice was most relevant since these mice express excess human ACE2 to which the SARS-CoV-2 spike protein RBD mainly binds [95]. Intriguingly, when the SARS-CoV-2 spike protein RBD binds to ACE-2 receptors, ACE2 receptors are internalized and degraded, which in turn can increase angiotensin II (Ag II) which tends to act on angiotensin receptor 1 (ATR1). The activation of ATR1 would activate NADPH oxidase (NOX), promoting the production of hydrogen peroxides, lipid peroxidation, and ferroptosis [96]. The implication of ACE2 receptors in the induction or mitigation of ferroptosis was repeatedly reported [97,98]. However, these mechanisms remain to be investigated.

Despite the variability in the molecular response, ultrastructural examination with transmission electron microscopy showed classical ferroptotic features, including cell membrane disruption, shrunken mitochondria, disrupted cristae, and translucent nuclei and cytoplasm in the hippocampus and prefrontal cortex suggesting failed protective mechanisms against ferroptosis [3,67]. These ultrastructural findings, limited to the hippocampus and prefrontal cortex, support the notion that S1 exposure is associated with persistent cellular injury patterns compatible with ferroptosis in these regions, even when biochemical markers vary by region and time point or fail to reach week-specific statistical significance after correction.

## 4. Methods

### 4.1. Mice

All studies were approved by the Mayo Clinic Institutional Animal Care and Use Committee under Protocol number A00006724-22-R25 (7 December 2022) and carried out in accordance with the National Institutes of Health Guide for the Care and Use of Laboratory Animals. We used a total of 38 male K18-hACE2 transgenic mice (B6.Cg-Tg (K18-ACE2) 2Prlmn/J) purchased from the Jackson Laboratory (Bar Harbor, ME, USA). Mice were group-housed, 4–5 mice per cage, with available food and water ad libitum, at a temperature of 23–25 °C and humidity of 50–60% with a 14 h/10 h light/dark cycle (light: 5:00–19:00, dark: 19:00–5:00).

### 4.2. SARS-CoV-2 Spike Protein Receptor Binding Domain (RBD) Subunit 1 (S1) Administration

**Figure 9:**
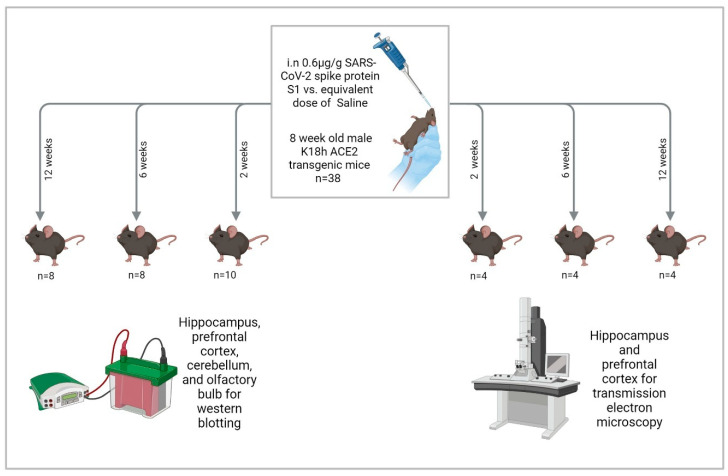
Study design. “Created in BioRender. Hatem, A. (2026) https://BioRender.com/qzdemai, accessed on 28 December 2025”.

We administered 0.6 μg/g intranasal SARS-CoV-2 spike protein subunit 1 (S1) (R&W Systems, Singapore, cat. no. 10522-CV) or an equivalent volume of phosphate-buffered saline (PBS) to mice once they reached 8 weeks of age. This dose is about 12 μg/20 g mouse, which is comparable to other studies [65,66,99]. S1 was reconstituted in PBS and administered to mice anesthetized with isoflurane (3–4% for induction and 1.5–2% for maintenance) in oxygen using a precision vaporizer. Reconstituted S1 was administered as 3 μL drops in the left nostril and 3 μL drops in the right nostril, separated by 15 s, then the same 2 × 3 μL drop administration was repeated after 30 s until the full dose had been administered [100]. Mice were euthanized at 2, 6, and 12 weeks after S1 administration by cervical dislocation followed by rapid decapitation, brain dissection on ice, and flash freezing of brain tissues in liquid nitrogen. We collected the olfactory bulbs, prefrontal cortices, hippocampi, and cerebellums bilaterally. Tissues were stored in a −80 °F freezer until they were assayed (Figure 9).

### 4.3. Western Blotting

Tissue extracts were isolated using RIPA lysis buffer (50 mM Tris-HCl, 150 mM NaCl, 10 mM EDTA, 1% Triton X-100), supplemented with protease and phosphatase inhibitors (Thermo Scientific, Waltham, MA, USA, cat. No. PIA32959). Total protein concentrations were quantified using Pierce BCA Protein Assay Kit (Thermo Scientific, Waltham, MA, USA, cat. No. 23225) following the manufacturer’s instructions. Equal amounts of protein (30 µg per sample) were resolved on 4–20% Mini-PROTEAN^®^ TGX™ precast protein gels (Bio-Rad, Hercules, CA, USA, cat. no. 4561096) and transferred onto PVDF membranes (Thermo Scientific, Waltham, MA, USA, cat. no. 88518). Membranes were blocked for 1 h at room temperature with blocking solution (Tris-buffered saline with 0.05% Tween (TBS-T) containing 5% skim milk), then incubated overnight at 4 °C with primary antibodies. We used primary antibodies for transferrin receptor 1 (TFR1) (Cell Signaling Technology, Danvers, MA, USA, Cat. no. 46222) (1:1000), ferroportin 1 (FPN1) (Proteintech, Chicago, IL, USA, Cat. no. 26601-1-AP) (1:2000), divalent metal transporter-1 (DMT-1) (Abcam, Washington, DC, USA, Cat. no. ab55735) (1:1000), MDA-conjugated proteins (Abcam, Washington, DC, USA, Cat. no. ab243066) (1:1000), Nuclear Factor Erythroid 2-Related Factor 2 (NrF2) (Abcam, Washington, DC, USA, Cat. no. ab62352) (1:2000), GPx4 (Cell Signaling Technology, Danvers, MA, USA, Cat. no. 52455) (1:1000), and beta-actin (Cell Signaling Technology, Danvers, MA, USA, Cat. no. 3700T) (1:1000). Following 3 washes with TBS-T, membranes were further incubated with either horseradish peroxidase (HRP)-conjugated goat anti-rabbit antibody (BioRad, Hercules, CA, USA, Cat. no. 1706515) (1:7500) or horseradish peroxidase (HRP)-conjugated goat anti-mouse antibody (VWR International LLC, Radnor, PA, USA, Cat. no. P001982153) (1:5000) in blocking solution for 2 h at room temperature. For the assessment of beta-actin expression, membranes were stripped with 0.2 M NaOH, washed, and incubated with beta-actin antibody using the same procedure as above. Blots were incubated in SuperSignal™ West Femto Maximum Sensitivity Substrate (Thermo Scientific, Waltham, MA, USA, cat. no. 34094). Bands were detected with a digital GE Amersham Imager 680. Band intensities were measured in arbitrary units using QuantityOne software V.4.6.8 (BioRad, Hercules, CA, USA). Band intensity values were normalized over the beta-actin band of the same lane. Relative protein expression was determined as the ratio of each sample over the average of controls.

Statistical analyses were performed unblinded using GraphPad Prism software (V.10.4.1, San Diego, CA, USA). For Western blot experiments, relative protein expression was analyzed using a two-way ANOVA, with factor treatment (saline vs. SARS-CoV-2 S1) and time (2, 6, and 12 weeks). When main effects and/or treatment × time interactions were detected, Šídák-adjusted multiple comparisons were performed to compare saline and spike groups within each time point. Data were presented as mean ± SEM. Results were considered statistically significant when *p* < 0.05.

### 4.4. Transmission Electron Microscopy

Mice were transcardially perfused with 4% paraformaldehyde (PFA). After perfusion, brains were dissected and both hippocampus and prefrontal cortex were cut into 2–4 mm^3^ pieces and placed into 4% paraformaldehyde + 1% glutaraldehyde (in 0.1 M phosphate buffer pH 7.4) fixative immediately. Then, tissues were post-fixed in osmium tetroxide, dehydrated, and embedded in resin. Ultrathin sections were cut, stained, and imaged on a JEOL 1400 transmission electron microscope (JEOL Ltd., Akishima, Tokyo, Japan) at an accelerating voltage of 80 kV.

## 5. Conclusions

Collectively, our results suggest that a single intranasal dose of the SARS-CoV-2 spike protein induces alterations of ferroptotic markers through temporal and region-specific changes in a targeted panel of iron-handling, antioxidant, and lipid peroxidation-related markers, alongside ultrastructural ferroptotic features in brain tissue. However, these results raise multiple questions, including the following: What are the clinical implications for such changes in the ferroptotic markers? What are the consequences for the dichotomy of ferroptosis and anti-ferroptotic compensatory mechanisms, especially since ferroptosis is implicated in multiple pathologies yet has beneficial outcomes in certain cases such as tumorigenesis [101]?

Several limitations should be considered. First, the sample size at each time point (n = 4–5) may reduce the power to detect week-specific post hoc differences even when ANOVA identifies significant time or interaction effects, especially with the unblinded nature of the analysis. Second, experimental design introduces potential observer bias, particularly in the selection of representative microscopy images and Western blot quantification, and limits the statistical power and generalizability of the findings. Future studies must validate these results in larger, blinded cohorts. Third, our marker panel captures key nodes of ferroptosis biology. However, more depth in investigating the ferroptosis machinery is needed by examining more ferroptosis-related markers and examining the effect of anti-ferroptosis agents such as ferrostatin-1. Fourth, Western blotting reflects bulk tissue protein levels and cannot resolve cell-type-specific effects. Fifth, ultrastructural examination of ferroptotic features was limited to the hippocampus and prefrontal cortex and depended on qualitative assessment rather than quantitative one. Therefore, future studies will benefit from expanding ferroptosis markers, incorporating anti-ferroptotic interventions (e.g., ferrostatin-1), assessing earlier and later time points. In addition, utilizing cell-type-resolved approaches such as single-cell/spatial transcriptomics or proteomics is needed to determine whether these region-specific signals arise from specific cell populations, which would better clarify our findings and uncover the underlying pathological mechanisms of the spike protein in relation to post-COVID syndrome.

## Data Availability

The original contributions presented in this study are included in the article/Supplementary Material, further inquiries can be directed to the corresponding author.

## Supplementary Materials

The following supporting information can be downloaded at: https://www.mdpi.com/article/10.3390/ijms27031526/s1.

## References

[1] Dixon S.J., Lemberg K.M., Lamprecht M.R., Skouta R., Zaitsev E.M., Gleason C.E., Patel D.N., Bauer A.J., Cantley A.M., Yang W.S. (2012). Ferroptosis: An iron-dependent form of nonapoptotic cell death. Cell.

[2] Dixon S.J., Stockwell B.R. (2019). The hallmarks of ferroptosis. Annu. Rev. Cancer Biol..

[3] Stockwell B.R. (2022). Ferroptosis turns 10: Emerging mechanisms, physiological functions, and therapeutic applications. Cell.

[4] Cao H., Zuo C., Huang Y., Zhu L., Zhao J., Yang Y., Jiang Y., Wang F. (2021). Hippocampal proteomic analysis reveals activation of necroptosis and ferroptosis in a mouse model of chronic unpredictable mild stress-induced depression. Behav. Brain Res..

[5] Dai Y., Guo J., Zhang B., Chen J., Ou H., He R.-R., So K.-F., Zhang L. (2023). Lycium barbarum (Wolfberry) glycopeptide prevents stress-induced anxiety disorders by regulating oxidative stress and ferroptosis in the medial prefrontal cortex. Phytomedicine.

[6] Yehia A., Melhuish Beaupre L.M., Ho M.C., Biernnacka J.M., Frye M.A., Abulseoud O.A. (2025). Ferroptosis as a potential molecular mechanism of bipolar disorder. Transl. Psychiatry.

[7] Cui Y., Zhang Y., Zhao X., Shao L., Liu G., Sun C., Xu R., Zhang Z. (2021). ACSL4 exacerbates ischemic stroke by promoting ferroptosis-induced brain injury and neuroinflammation. Brain Behav. Immun..

[8] Yang K., Zeng L., Yuan X., Wang S., Ge A., Xu H., Zeng J., Ge J. (2022). The mechanism of ferroptosis regulating oxidative stress in ischemic stroke and the regulation mechanism of natural pharmacological active components. Biomed. Pharmacother..

[9] Kenny E.M., Fidan E., Yang Q., Anthonymuthu T.S., New L.A., Meyer E.A., Wang H., Kochanek P.M., Dixon C.E., Kagan V.E. (2019). Ferroptosis contributes to neuronal death and functional outcome after traumatic brain injury. Crit. Care Med..

[10] Xie B.S., Wang Y.Q., Lin Y., Mao Q., Feng J.F., Gao G.Y., Jiang J.Y. (2019). Inhibition of ferroptosis attenuates tissue damage and improves long-term outcomes after traumatic brain injury in mice. CNS Neurosci. Ther..

[11] Bao W.-D., Pang P., Zhou X.-T., Hu F., Xiong W., Chen K., Wang J., Wang F., Xie D., Hu Y.-Z. (2021). Loss of ferroportin induces memory impairment by promoting ferroptosis in Alzheimer’s disease. Cell Death Differ..

[12] He Y.-J., Cong L., Liang S.-L., Ma X., Tian J.-N., Li H., Wu Y. (2022). Discovery and validation of Ferroptosis-related molecular patterns and immune characteristics in Alzheimer’s disease. Front. Aging Neurosci..

[13] Park M.W., Cha H.W., Kim J., Kim J.H., Yang H., Yoon S., Boonpraman N., Yi S.S., Yoo I.D., Moon J.-S. (2021). NOX4 promotes ferroptosis of astrocytes by oxidative stress-induced lipid peroxidation via the impairment of mitochondrial metabolism in Alzheimer’s diseases. Redox Biol..

[14] Do Van B., Gouel F., Jonneaux A., Timmerman K., Gelé P., Pétrault M., Bastide M., Laloux C., Moreau C., Bordet R. (2016). Ferroptosis, a newly characterized form of cell death in Parkinson’s disease that is regulated by PKC. Neurobiol. Dis..

[15] Tian Y., Lu J., Hao X., Li H., Zhang G., Liu X., Li X., Zhao C., Kuang W., Chen D. (2020). FTH1 inhibits ferroptosis through ferritinophagy in the 6-OHDA model of Parkinson’s disease. Neurotherapeutics.

[16] Song X., Wang Z., Tian Z., Wu M., Zhou Y., Zhang J. (2023). Identification of Key Ferroptosis-Related Genes in the Peripheral Blood of Patients with Relapsing-Remitting Multiple Sclerosis and Its Diagnostic Value. Int. J. Mol. Sci..

[17] Van San E., Debruyne A.C., Veeckmans G., Tyurina Y.Y., Tyurin V.A., Zheng H., Choi S.M., Augustyns K., van Loo G., Michalke B. (2023). Ferroptosis contributes to multiple sclerosis and its pharmacological targeting suppresses experimental disease progression. Cell Death Differ..

[18] Wang D., Liang W., Huo D., Wang H., Wang Y., Cong C., Zhang C., Yan S., Gao M., Su X. (2023). SPY1 inhibits neuronal ferroptosis in amyotrophic lateral sclerosis by reducing lipid peroxidation through regulation of GCH1 and TFR1. Cell Death Differ..

[19] Wang T., Tomas D., Perera N.D., Cuic B., Luikinga S., Viden A., Barton S.K., McLean C.A., Samson A.L., Southon A. (2022). Ferroptosis mediates selective motor neuron death in amyotrophic lateral sclerosis. Cell Death Differ..

[20] Yehia A., Abulseoud O.A. (2024). Melatonin: A ferroptosis inhibitor with potential therapeutic efficacy for the post-COVID-19 trajectory of accelerated brain aging and neurodegeneration. Mol. Neurodegener..

[21] Jennings G., Monaghan A., Xue F., Mockler D., Romero-Ortuno R. (2021). A Systematic Review of Persistent Symptoms and Residual Abnormal Functioning following Acute COVID-19: Ongoing Symptomatic Phase vs. Post-COVID-19 Syndrome. J. Clin. Med..

[22] Carod-Artal F.J. (2021). Post-COVID-19 syndrome: Epidemiology, diagnostic criteria and pathogenic mechanisms involved. Rev. Neurol..

[23] Raveendran A.V., Jayadevan R., Sashidharan S. (2021). Long COVID: An overview. Diabetes Metab. Syndr..

[24] Meo S.A., Abukhalaf A.A., Alomar A.A., Al-Hussain F. (2021). Magnetic Resonance Imaging (MRI) and neurological manifestations in SARS-CoV-2 patients. Eur. Rev. Med. Pharmacol. Sci..

[25] Anjana N.K.N., Annie T.T., Siba S., Meenu M.S., Chintha S., Anish T.S.N. (2021). Manifestations and risk factors of post COVID syndrome among COVID-19 patients presented with minimal symptoms—A study from Kerala, India. J. Fam. Med. Prim. Care.

[26] Taquet M., Geddes J.R., Husain M., Luciano S., Harrison P.J. (2021). 6-month neurological and psychiatric outcomes in 236 379 survivors of COVID-19: A retrospective cohort study using electronic health records. Lancet Psychiatry.

[27] Hastie C.E., Lowe D.J., McAuley A., Mills N.L., Winter A.J., Black C., Scott J.T., O’donnell C.A., Blane D.N., Browne S. (2023). True prevalence of long-COVID in a nationwide, population cohort study. Nat. Commun..

[28] Davis H.E., McCorkell L., Vogel J.M., Topol E.J. (2023). Long COVID: Major findings, mechanisms and recommendations. Nat. Rev. Microbiol..

[29] Sousa R.A., Yehia A., Abulseoud O.A. (2023). Attenuation of ferroptosis as a potential therapeutic target for neuropsychiatric manifestations of post-COVID syndrome. Front. Neurosci..

[30] Maio N., Lafont B.A., Sil D., Li Y., Bollinger J.M., Krebs C., Pierson T.C., Linehan W.M., Rouault T.A. (2021). Fe-S cofactors in the SARS-CoV-2 RNA-dependent RNA polymerase are potential antiviral targets. Science.

[31] Abulseoud O.A., Yehia A., Egol C.J., Nettey V.N., Aly M., Qu Y., Skolnik A.B., Grill M.F., Sen A., Schneekloth T.D. (2022). Attenuated initial serum ferritin concentration in critically ill coronavirus disease 2019 geriatric patients with comorbid psychiatric conditions. Front. Psychiatry.

[32] Jia F., Liu H., Kang S. (2021). NCOA4-mediated ferritinophagy: A vicious culprit in COVID-19 pathogenesis?. Front. Mol. Biosci..

[33] Kaushal K., Kaur H., Sarma P., Bhattacharyya A., Sharma D.J., Prajapat M., Pathak M., Kothari A., Kumar S., Rana S. (2022). Serum ferritin as a predictive biomarker in COVID-19. A systematic review, meta-analysis and meta-regression analysis. J. Crit. Care.

[34] Lin Z., Long F., Yang Y., Chen X., Xu L., Yang M. (2020). Serum ferritin as an independent risk factor for severity in COVID-19 patients. J. Infect..

[35] Qeadan F., Tingey B., Gu L.Y., Packard A.H., Erdei E., Saeed A.I. (2021). Prognostic values of serum ferritin and D-dimer trajectory in patients with COVID-19. Viruses.

[36] Cheng L., Li H., Li L., Liu C., Yan S., Chen H., Li Y. (2020). Ferritin in the coronavirus disease 2019 (COVID-19): A systematic review and meta-analysis. J. Clin. Lab. Anal..

[37] Zhao K., Huang J., Dai D., Feng Y., Liu L., Nie S. (2020). Serum iron level as a potential predictor of coronavirus disease 2019 severity and mortality: A retrospective study. Proceedings of the Open Forum Infectious Diseases.

[38] Hanson A.L., Mulè M.P., Ruffieux H., Mescia F., Bergamaschi L., Pelly V.S., Turner L., Kotagiri P., Cambridge Institute of Therapeutic Immunology, Infectious Disease–National Institute for Health Research (CITIID–NIHR) COVID BioResource Collaboration (2024). Iron dysregulation and inflammatory stress erythropoiesis associates with long-term outcome of COVID-19. Nat. Immunol..

[39] Karkhanei B., Ghane E.T., Mehri F. (2021). Evaluation of oxidative stress level: Total antioxidant capacity, total oxidant status and glutathione activity in patients with COVID-19. New Microbes New Infect..

[40] Kumar P., Osahon O., Vides D.B., Hanania N., Minard C.G., Sekhar R.V. (2021). Severe glutathione deficiency, oxidative stress and oxidant damage in adults hospitalized with COVID-19: Implications for GlyNAC (Glycine and N-Acetylcysteine) supplementation. Antioxidants.

[41] Muhammad Y., Kani Y.A., Iliya S., Muhammad J.B., Binji A., El-Fulaty Ahmad A., Kabir M.B., Umar Bindawa K., Ahmed A.u. (2021). Deficiency of antioxidants and increased oxidative stress in COVID-19 patients: A cross-sectional comparative study in Jigawa, Northwestern Nigeria. SAGE Open Med..

[42] Pincemail J., Cavalier E., Charlier C., Cheramy–Bien J.-P., Brevers E., Courtois A., Fadeur M., Meziane S., Goff C.L., Misset B. (2021). Oxidative stress status in COVID-19 patients hospitalized in intensive care unit for severe pneumonia. A pilot study. Antioxidants.

[43] Çakırca G., Damar Çakırca T., Üstünel M., Torun A., Koyuncu I. (2021). Thiol level and total oxidant/antioxidant status in patients with COVID-19 infection. Ir. J. Med. Sci. (1971-).

[44] Polonikov A. (2020). Endogenous deficiency of glutathione as the most likely cause of serious manifestations and death in COVID-19 patients. ACS Infect. Dis..

[45] Wang Y., Huang J., Sun Y., Stubbs D., He J., Li W., Wang F., Liu Z., Ruzicka J.A., Taylor E.W. (2021). SARS-CoV-2 suppresses mRNA expression of selenoproteins associated with ferroptosis, endoplasmic reticulum stress and DNA synthesis. Food Chem. Toxicol..

[46] Moghaddam A., Heller R.A., Sun Q., Seelig J., Cherkezov A., Seibert L., Hackler J., Seemann P., Diegmann J., Pilz M. (2020). Selenium deficiency is associated with mortality risk from COVID-19. Nutrients.

[47] Younesian O., Khodabakhshi B., Abdolahi N., Norouzi A., Behnampour N., Hosseinzadeh S., Alarzi S.S.H., Joshaghani H. (2021). Decreased serum selenium levels of COVID-19 patients in comparison with healthy individuals. Biol. Trace Elem. Res..

[48] Ingold I., Berndt C., Schmitt S., Doll S., Poschmann G., Buday K., Roveri A., Peng X., Freitas F.P., Seibt T. (2018). Selenium utilization by GPX4 is required to prevent hydroperoxide-induced ferroptosis. Cell.

[49] Ursini F., Maiorino M. (2020). Lipid peroxidation and ferroptosis: The role of GSH and GPx4. Free Radic. Biol. Med..

[50] Al-Hakeim H.K., Al-Rubaye H.T., Al-Hadrawi D.S., Almulla A.F., Maes M. (2023). Long-COVID post-viral chronic fatigue and affective symptoms are associated with oxidative damage, lowered antioxidant defenses and inflammation: A proof of concept and mechanism study. Mol. Psychiatry.

[51] Saleh M.G., Chang L., Liang H., Ryan M.C., Cunningham E., Garner J., Wilson E., Levine A.R., Kottilil S., Ernst T. (2023). Ongoing oxidative stress in individuals with post-acute sequelae of COVID-19. Neuroimmune Pharmacol. Ther..

[52] Poletti S., Paolini M., Mazza M.G., Palladini M., Furlan R., Querini P.R., Benedetti F., Covid BioB Outpatients Clinic Study Group (2022). Lower levels of glutathione in the anterior cingulate cortex associate with depressive symptoms and white matter hyperintensities in COVID-19 survivors. Eur. Neuropsychopharmacol..

[53] Martín-Fernández M., Aller R., Heredia-Rodríguez M., Gómez-Sánchez E., Martínez-Paz P., Gonzalo-Benito H., Sánchez-de Prada L., Gorgojo Ó., Carnicero-Frutos I., Tamayo E. (2021). Lipid peroxidation as a hallmark of severity in COVID-19 patients. Redox Biol..

[54] Žarković N., Orehovec B., Milković L., Baršić B., Tatzber F., Wonisch W., Tarle M., Kmet M., Mataić A., Jakovčević A. (2021). Preliminary Findings on the Association of the Lipid Peroxidation Product 4-Hydroxynonenal with the Lethal Outcome of Aggressive COVID-19. Antioxidants.

[55] Zarkovic N., Jakovcevic A., Mataic A., Jaganjac M., Vukovic T., Waeg G., Zarkovic K. (2022). Post-mortem findings of inflammatory cells and the association of 4-hydroxynonenal with systemic vascular and oxidative stress in lethal COVID-19. Cells.

[56] Caterino M., Gelzo M., Sol S., Fedele R., Annunziata A., Calabrese C., Fiorentino G., D’Abbraccio M., Dell’Isola C., Fusco F.M. (2021). Dysregulation of lipid metabolism and pathological inflammation in patients with COVID-19. Sci. Rep..

[57] Žarković N., Łuczaj W., Jarocka-Karpowicz I., Orehovec B., Baršić B., Tarle M., Kmet M., Lukšić I., Biernacki M., Skrzydlewska E. (2022). Diversified Effects of COVID-19 as a Consequence of the Differential Metabolism of Phospholipids and Lipid Peroxidation Evaluated in the Plasma of Survivors and Deceased Patients upon Admission to the Hospital. Int. J. Mol. Sci..

[58] López-Hernández Y., Oropeza-Valdez J.J., García Lopez D.A., Borrego J.C., Murgu M., Valdez J., López J.A., Monárrez-Espino J. (2023). Untargeted analysis in post-COVID-19 patients reveals dysregulated lipid pathways two years after recovery. Front. Mol. Biosci..

[59] Garrido P., De los Santos Castillo-Peinado L., Priego-Capote F., Barrio I., Piñeiro Á., Domínguez-Santalla M., Rodríguez-Ruiz E., Garcia-Fandino R. (2024). Lipidomics signature in post-COVID patient sera and its influence on the prolonged inflammatory response. J. Infect. Public Health.

[60] Yang W.S., Stockwell B.R. (2016). Ferroptosis: Death by lipid peroxidation. Trends Cell Biol..

[61] Rodencal J., Dixon S.J. (2023). A tale of two lipids: Lipid unsaturation commands ferroptosis sensitivity. Proteomics.

[62] Winkler E.S., Bailey A.L., Kafai N.M., Nair S., McCune B.T., Yu J., Fox J.M., Chen R.E., Earnest J.T., Keeler S.P. (2020). SARS-CoV-2 infection of human ACE2-transgenic mice causes severe lung inflammation and impaired function. Nat. Immunol..

[63] Cosentino M., Marino F. (2022). Understanding the pharmacology of COVID-19 mRNA vaccines: Playing dice with the spike?. Int. J. Mol. Sci..

[64] Swank Z., Senussi Y., Manickas-Hill Z., Yu X.G., Li J.Z., Alter G., Walt D.R. (2023). Persistent circulating severe acute respiratory syndrome coronavirus 2 spike is associated with post-acute coronavirus disease 2019 sequelae. Clin. Infect. Dis..

[65] Fontes-Dantas F.L., Fernandes G.G., Gutman E.G., De Lima E.V., Antonio L.S., Hammerle M.B., Mota-Araujo H.P., Colodeti L.C., Araujo S.M.B., Froz G.M. (2023). SARS-CoV-2 Spike protein induces TLR4-mediated long-term cognitive dysfunction recapitulating post-COVID-19 syndrome in mice. Cell Rep..

[66] Burnett F.N., Coucha M., Bolduc D.R., Hermanns V.C., Heath S.P., Abdelghani M., Macias-Moriarity L.Z., Abdelsaid M. (2023). SARS-CoV-2 Spike Protein Intensifies Cerebrovascular Complications in Diabetic hACE2 Mice through RAAS and TLR Signaling Activation. Int. J. Mol. Sci..

[67] Miyake S., Murai S., Kakuta S., Uchiyama Y., Nakano H. (2020). Identification of the hallmarks of necroptosis and ferroptosis by transmission electron microscopy. Biochem. Biophys. Res. Commun..

[68] Youdim M.B. (2008). Brain iron deficiency and excess; cognitive impairment and neurodegeneration with involvement of striatum and hippocampus. Neurotox. Res..

[69] Nouraeinejad A. (2025). Memory loss in patients with long COVID can be due to reduced hippocampal neurogenesis. Eur. Arch. Psychiatry Clin. Neurosci..

[70] Keklicek H., Selçuk H., Kurt İ., Ulukaya S., Öztürk G. (2022). Individuals with a COVID-19 history exhibit asymmetric gait patterns despite full recovery. J. Biomech..

[71] Xydakis M.S., Albers M.W., Holbrook E.H., Lyon D.M., Shih R.Y., Frasnelli J.A., Pagenstecher A., Kupke A., Enquist L.W., Perlman S. (2021). Post-viral effects of COVID-19 in the olfactory system and their implications. Lancet Neurol..

[72] Kandemirli S.G., Altundag A., Yildirim D., Tekcan Sanli D.E., Saatci O. (2021). Olfactory Bulb MRI and Paranasal Sinus CT Findings in Persistent COVID-19 Anosmia. Acad. Radiol..

[73] Feng H., Schorpp K., Jin J., Yozwiak C.E., Hoffstrom B.G., Decker A.M., Rajbhandari P., Stokes M.E., Bender H.G., Csuka J.M. (2020). Transferrin Receptor Is a Specific Ferroptosis Marker. Cell Rep..

[74] Park E., Chung S.W. (2019). ROS-mediated autophagy increases intracellular iron levels and ferroptosis by ferritin and transferrin receptor regulation. Cell Death Dis..

[75] Wu Y., Jiao H., Yue Y., He K., Jin Y., Zhang J., Zhang J., Wei Y., Luo H., Hao Z. (2022). Ubiquitin ligase E3 HUWE1/MULE targets transferrin receptor for degradation and suppresses ferroptosis in acute liver injury. Cell Death Differ..

[76] Hiromatsu M., Toshida K., Itoh S., Harada N., Kohashi K., Oda Y., Yoshizumi T. (2023). Transferrin Receptor is Associated with Sensitivity to Ferroptosis Inducers in Hepatocellular Carcinoma. Ann. Surg. Oncol..

[77] Wang X., Wen Z., Cao H., Luo J., Shuai L., Wang C., Ge J., Wang X., Bu Z., Wang J. (2023). Transferrin receptor protein 1 cooperates with mGluR2 to mediate the internalization of rabies virus and SARS-CoV-2. J. Virol..

[78] Zhang H., Ostrowski R., Jiang D., Zhao Q., Liang Y., Che X., Zhao J., Xiang X., Qin W., He Z. (2021). Hepcidin Promoted Ferroptosis through Iron Metabolism which Is Associated with DMT1 Signaling Activation in Early Brain Injury following Subarachnoid Hemorrhage. Oxid. Med. Cell. Longev..

[79] Song Q., Peng S., Sun Z., Heng X., Zhu X. (2021). Temozolomide Drives Ferroptosis via a DMT1-Dependent Pathway in Glioblastoma Cells. Yonsei Med. J..

[80] Peng W., Chung K.B., Lawrence B.P., O’Banion M.K., Dirksen R.T., Wojtovich A.P., Onukwufor J.O. (2024). DMT1 knockout abolishes ferroptosis induced mitochondrial dysfunction in *C. elegans* amyloid β proteotoxicity. Free Radic. Biol. Med..

[81] Li D., Chen Y., Zhang B., Heng X., Yin J., Zhao P., Sun N., Shao C. (2025). Praeruptorin A screened by a ferrous ion probe inhibited DMT1 and ferroptosis to attenuate Doxorubicin-induced cardiomyopathy. Eur. J. Med. Chem..

[82] Shi J., Yang M.M., Yang S., Fan F., Zheng G., Miao Y., Hua Y., Zhang J., Cheng Y., Liu S. (2024). MaiJiTong granule attenuates atherosclerosis by reducing ferroptosis via activating STAT6-mediated inhibition of DMT1 and SOCS1/p53 pathways in LDLR(-/-) mice. Phytomedicine.

[83] Jiang S., Shi X., Kong X., Chen Y., Xu W., Hao M., Wei D., Gao F., Wang F., Pu W. (2025). Iron dysregulation and ferroptosis are associated with pulmonary fibrosis: Insight from idiopathic pulmonary fibrosis, systemic sclerosis, and COVID-19 patients. J. Trace Elem. Med. Biol..

[84] Ferreira H.B., Domingues M.R. (2024). Oxidized phospholipid-protein adducts: The future targets of interest. Arch. Biochem. Biophys..

[85] Zhu S., Li W., Hao Y., Zhang L., Gao P. (2025). TIPE2 suppresses ferroptosis and pro-inflammatory polarization in macrophages triggered by SARS-CoV-2 spike protein. Sci. Rep..

[86] Nguyen V., Zhang Y., Gao C., Cao X., Tian Y., Carver W., Kiaris H., Cui T., Tan W. (2022). The spike protein of sars-cov-2 impairs lipid metabolism and increases susceptibility to lipotoxicity: Implication for a role of nrf2. Cells.

[87] Dedoni S., Avdoshina V., Camoglio C., Siddi C., Fratta W., Scherma M., Fadda P. (2022). K18-and CAG-hACE2 Transgenic Mouse Models and SARS-CoV-2: Implications for Neurodegeneration Research. Molecules.

[88] Pandey K., Acharya A., Mohan M., Ng C.L., Reid S.P., Byrareddy S.N. (2021). Animal models for SARS-CoV-2 research: A comprehensive literature review. Transbound. Emerg. Dis..

[89] McCray P.B., Pewe L., Wohlford-Lenane C., Hickey M., Manzel L., Shi L., Netland J., Jia H.P., Halabi C., Sigmund C.D. (2007). Lethal infection of K18-hACE2 mice infected with severe acute respiratory syndrome coronavirus. J. Virol..

[90] Oladunni F.S., Park J.-G., Pino P.A., Gonzalez O., Akhter A., Allué-Guardia A., Olmo-Fontánez A., Gautam S., Garcia-Vilanova A., Ye C. (2020). Lethality of SARS-CoV-2 infection in K18 human angiotensin-converting enzyme 2 transgenic mice. Nat. Commun..

[91] Golden J.W., Cline C.R., Zeng X., Garrison A.R., Carey B.D., Mucker E.M., White L.E., Shamblin J.D., Brocato R.L., Liu J. (2020). Human angiotensin-converting enzyme 2 transgenic mice infected with SARS-CoV-2 develop severe and fatal respiratory disease. JCI Insight.

[92] Rhea E.M., Logsdon A.F., Hansen K.M., Williams L.M., Reed M.J., Baumann K.K., Holden S.J., Raber J., Banks W.A., Erickson M.A. (2021). The S1 protein of SARS-CoV-2 crosses the blood–brain barrier in mice. Nat. Neurosci..

[93] de Melo B.P., da Silva J.A.M., Rodrigues M.A., Palmeira J.d.F., Saldanha-Araujo F., Argañaraz G.A., Argañaraz E.R. (2025). SARS-CoV-2 Spike Protein and Long COVID—Part 1: Impact of Spike Protein in Pathophysiological Mechanisms of Long COVID Syndrome. Viruses.

[94] Hoffmann M., Kleine-Weber H., Pöhlmann S. (2020). A multibasic cleavage site in the spike protein of SARS-CoV-2 is essential for infection of human lung cells. Mol. Cell.

[95] Jackson C.B., Farzan M., Chen B., Choe H. (2022). Mechanisms of SARS-CoV-2 entry into cells. Nat. Rev. Mol. Cell Biol..

[96] Fratta Pasini A.M., Stranieri C., Girelli D., Busti F., Cominacini L. (2021). Is Ferroptosis a Key Component of the Process Leading to Multiorgan Damage in COVID-19?. Antioxidants.

[97] Abudukeremu A., Aikemu A., Yang T., Fang L., Shanahati D., Nijiati Y. (2025). Mechanism of ferroptosis in hypoxia-induced pulmonary vascular remodeling in hypoxia pulmonary hypertension: A study based on the ACE2-Ang-(1-7)-Mas axis. Chem. Biol. Interact..

[98] Wu G., Ma C., Xu J., Chen X., Chen X., Wang G., Zhang Y. (2026). ACE2 mitigates Streptococcus uberis-induced ferroptosis in goat mammary epithelial cells by inhibiting ROS-chaperone-mediated autophagic degradation of GPX4. Microb. Pathog..

[99] Oh J., Cho W.-H., Barcelon E., Kim K.H., Hong J., Lee S.J. (2022). SARS-CoV-2 spike protein induces cognitive deficit and anxiety-like behavior in mouse via non-cell autonomous hippocampal neuronal death. Sci. Rep..

[100] Hanson L.R., Fine J.M., Svitak A.L., Faltesek K.A. (2013). Intranasal administration of CNS therapeutics to awake mice. J. Vis. Exp. JoVE.

[101] Zhou Q., Meng Y., Li D., Yao L., Le J., Liu Y., Sun Y., Zeng F., Chen X., Deng G. (2024). Ferroptosis in cancer: From molecular mechanisms to therapeutic strategies. Signal Transduct. Target. Ther..

