# Supplementary material for: SARS-CoV-2 Spike Protein Induces Time-Dependent and Brain-Region-Specific Alterations in Ferroptosis Markers: A Preliminary Study in K18-hACE2 Mice
Source: Int J Mol Sci. 2026 Feb 4;27(3):1526. doi: 10.3390/ijms27031526 (PMC12897609; doi:10.3390/ijms27031526)
Supplement: Supplementary file 1 [file ijms-27-01526-s001.zip › ijms-4093240-supplementary.pdf]

Supplementary tables:

Table 1s: Hippocampal changes in the expression of ferroptotic markers (two-way ANOVA)

Hippocampus							
		Main effect			Pairwise analysis		
		treatment	time	Treatment*time	2WK	6WK	12WK
TFR1	*p* value	0.1585	0.028*	0.0183*	0.7359	0.074	0.4297
	F (DFn, DFd)	F (1, 19) = 2.155	F (1.584, 15.05) = 4.972	F (2, 19) = 4.972			
FPN1	*p* value	0.4315	0.0102*	0.0113*	0.0126*	0.4489	0.9853
	F (DFn, DFd)	F (1, 7) = 0.6965	F (2, 12) = 6.879	F (2, 12) = 6.666			
DMT1	*p* value	0.5562	0.519	0.2753	0.5007	0.7147	0.8538
	F (DFn, DFd)	F (1, 10) = 0.3707	F (2, 10) = 0.7009	F (2, 10) = 1.471			
NrF2	*p* value	0.0796	0.1077	0.3804	0.3029	0.3923	>0.9999
	F (DFn, DFd)	F (1, 10) = 3.807	F (2, 10) = 2.808	F (2, 10) = 1.066			
GPx4	*p* value	0.2949	0.6121	0.3665	0.9998	0.2709	0.999
	F (DFn, DFd)	F (1, 10) = 1.222	F (2, 10) = 0.5157	F (2, 10) = 1.112			
MDA	*p* value	0.4906	0.8637	0.7701	0.7327	0.9958	>0.9999
	F (DFn, DFd)	F (1, 10) = 0.5120	F (2, 10) = 0.1487	F (2, 10) = 0.2682			

Table 2s: Prefrontal cortex changes in the expression of ferroptotic markers (two-way ANOVA)

Prefrontal cortex							
		Main effect			Pairwise analysis		
		treatment	time	treatment*time	2WK	6WK	12WK
TFR1	*p* value	0.2467	0.6315	0.7108	0.9872	0.5228	0.9657
	F (DFn, DFd)	F (1, 20) = 1.424	F (1.359, 13.59) = 0.3473	F (2, 20) = 0.3473			
FPN1	*p* value	0.1697	0.1482	0.1329	0.1801	0.8933	0.7835
	F (DFn, DFd)	F (1, 8) = 2.278	F (1.594, 9.562) = 2.399	F (2, 12) = 2.399			
DMT1	*p* value	0.002*	0.0001*	<0.0001*	0.0027*	0.8933	0.9939
	F (DFn, DFd)	F (1, 20) = 12.61	F (1.541, 15.41) = 20.12	F (2, 20) = 20.12			
NrF2	*p* value	0.9636	0.8001	0.8296	>0.9999	0.912	0.9912
	F (DFn, DFd)	F (1, 8) = 0.002219	F (1.698, 10.19) = 0.1849	F (2, 12) = 0.1897			
GPx4	*p* value	0.0015*	0.0582	0.058	0.4306	0.0527	0.8186
	F (DFn, DFd)	F (1, 20) = 13.60	F (1.994, 19.94) = 3.294	F (2, 20) = 3.294			
MDA	*p* value	0.5991	0.0152*	0.0111*	0.3114	0.6597	0.1448
	F (DFn, DFd)	F (1, 8) = 0.2995	F (1.858, 11.15) = 6.411	F (2, 12) = 6.712			

Table 3s: Cerebellar changes in the expression of ferroptotic markers (two-way ANOVA)

Cerebellum							
		Main effect			Pairwise analysis		
		treatment	time	treatment*time	2WK	6WK	12WK
TFR1	*p* value	0.003*	0.0045*	0.0023*	0.0545	0.3601	0.3273
	F (DFn, DFd)	F (1, 20) = 11.44	F (1.654, 16.54) = 8.343	F (2, 20) = 8.343			
FPN1	*p* value	0.0211*	0.0315*	0.0272*	0.0219*	0.999	0.9845
	F (DFn, DFd)	F (1, 20) = 6.266	F (1.829, 18.29) = 4.339	F (2, 20) = 4.339			
DMT1	*p* value	0.0364*	0.6223	0.6556	0.4836	0.6469	0.0684
	F (DFn, DFd)	F (1, 8) = 6.296	F (1.633, 9.799) = 0.4319	F (2, 12) = 0.4374			
NrF2	*p* value	0.4911	0.2942	0.2949	0.809	0.4758	0.8674
	F (DFn, DFd)	F (1, 20) = 0.4921	F (1.808, 18.08) = 1.299	F (2, 20) = 1.299			
GPx4	*p* value	0.0077*	0.0008*	0.0006*	0.0033*	0.3156	0.984
	F (DFn, DFd)	F (1, 8) = 12.47	F (1.842, 11.05) = 15.17	F (2, 12) = 14.87			
MDA	*p* value	0.7023	0.004*	0.0021*	0.5174	0.7694	0.043*
	F (DFn, DFd)	F (1, 8) = 0.1569	F (1.830, 10.98) = 9.883	F (2, 12) = 10.74			

Table 4s: olfactory bulb changes in the expression of ferroptotic markers (two-way ANOVA)

Olfactory bulb							
		Main effect			Pairwise analysis		
		treatment	time	treatment*time	2WK	6WK	12WK
TFR1	*p* value	0.6606	0.0014*	0.0046*	0.1207	>0.9999	0.542
	F (DFn, DFd)	F (1, 8) = 0.2078	F (1.998, 11.99) = 11.93	F (2, 12) = 8.720			
FPN1	*p* value	0.0203*	0.5625	0.6562	0.6224	0.5067	0.0006*
	F (DFn, DFd)	F (1, 8) = 8.329	F (1.149, 6.893) = 0.4261	F (2, 12) = 0.4364			
DMT1	*p* value	0.024*	0.8583	0.9276	0.5715	0.2189	0.6148
	F (DFn, DFd)	F (1, 20) = 5.962	F (1.356, 13.56) = 0.07548	F (2, 20) = 0.07548			
NrF2	*p* value	0.5271	0.6974	0.8414	0.9649	0.9994	0.6912
	F (DFn, DFd)	F (1, 20) = 0.4144	F (1.052, 10.52) = 0.1742	F (2, 20) = 0.1742			
GPx4	*p* value	0.0219*	0.208	0.1775	0.0799	0.6319	0.4121
	F (DFn, DFd)	F (1, 8) = 8.051	F (1.526, 9.155) = 1.876	F (2, 12) = 2.004			
MDA	*p* value	0.388	0.408	0.4513	0.8488	0.8511	0.7638
	F (DFn, DFd)	F (1, 20) = 0.7787	F (1.272, 12.72) = 0.8281	F (2, 20) = 0.8281			
